# Tuning the Structure and Photoluminescence of [SbCl_5_]^2−^-Based Halides via Modification of Imidazolium-Based Cations

**DOI:** 10.3390/molecules30163431

**Published:** 2025-08-20

**Authors:** Guoyang Chen, Xinping Guo, Haowei Lin, Zhizhuan Zhang, Abdusalam Ablez, Yuwei Ren, Kezhao Du, Xiaoying Huang

**Affiliations:** 1 College of Chemistry, Fuzhou University, Fuzhou 350108, China; chenguoyang@fjirsm.ac.cn (G.C.); guoxinping@fjirsm.ac.cn (X.G.); abdslm@fjirsm.ac.cn (A.A.); renyuwei@fjirsm.ac.cn (Y.R.); 2 State Key Laboratory of Structural Chemistry, Fujian Institute of Research on the Structure of Matter, Chinese Academy of Sciences, Fuzhou 350002, China; linhw@fjirsm.ac.cn (H.L.); zhangzhizhuan@stu.scu.edu.cn (Z.Z.); 3 Fujian College, University of Chinese Academy of Sciences, Fuzhou 100049, China; 4 Fujian Provincial Key Laboratory of Advanced Materials Oriented Chemical Engineering, Fujian Normal University, Fuzhou 350007, China

**Keywords:** imidazole-based ionic liquid, antimony halide, alkyl chain length, substituent, structural distortion

## Abstract

Structure–property relationships in imidazolium-based hybrid Sb(III) chlorides provide critical guidance for designing high-performance materials. Three zero-dimensional metal halides, namely, [C_3_mmim]_2_SbCl_5_ (**1**, [C_3_mmim]^+^ = 1-propyl-2,3-dimethylimidazolium), [C_5_mmim]_2_SbCl_5_ (**2**, [C_5_mmim]^+^ = 1-pentyl-2,3-dimethylimidazolium), and [C_5_mim]_2_SbCl_5_ (**3**, [C_5_mim]^+^ = 1-pentyl-3-methylimidazolium), are synthesized by ionothermal methods. These compounds exhibit markedly distinctly photophysical properties at their optimal excitation wavelengths. Structural analyses reveal that elongated alkyl chains in compounds **2** and **3** increase Sb–Sb distances compared to that in **1**, effectively isolating [SbCl_5_]^2−^ units, suppressing inter-center energy transfer, and reducing non-radiative transitions, thereby enhancing the photoluminescence quantum yield (PLQY). Furthermore, methyl substitution at the C2-position of the imidazolium ring in compounds **1** and **2** induces asymmetric coordination environments around the [SbCl_5_]^2−^ emission centers, leading to pronounced structural distortion. This distortion promotes non-radiative decay pathways and diminishes luminescent efficiency. Furthermore, temperature-dependent spectroscopy analysis and fitting of the Huang–Rhys factor (*S*) reveal significant electron–phonon coupling in compounds **1**–**3**, which effectively promotes the formation of self-trapped excitons (STEs). However, compound **1** exhibits extremely high *S*, which significantly enhances phonon-mediated non-radiative decay and ultimately reduces its PLQY. Overall, compound **3** has the highest PLQYs.

## 1. Introduction

Organic–inorganic metal halides (OIMHs) exhibit excellent luminescent properties, rendering them highly promising for applications in solid-state lighting [1,2], optical anti-counterfeiting [3], radiation detection [4,5], X-ray scintillation [6,7,8], and related fields. In recent years, ionic liquids have emerged as a preferred source of the organic cation components in OIMHs. This preference stems from their flexible cation selection (e.g., pyridinium, piperidinium, and imidazolium) and diverse anion options (e.g., Cl^−^ and Br^−^) [9]. Utilizing ionic liquid cations as organic cations, researchers have developed various low-dimensional luminescent metal halides, incorporating elements such as tin (Sn^2+^) [10], copper (Cu^+^), antimony (Sb^3+^) [11], zinc (Zn^2+^), and manganese (Mn^2+^). Among these, Sb^3+^ coordinates with halide ions (*X* = Cl, Br) to form diverse haloantimonate(III) anions, including [Sb*X*_4_]^−^ [12], [Sb*X*_5_]^2−^ [2,13,14,15], [Sb*X*_6_]^3−^ [16,17], [Sb_2_*X*_7_]^−^ [18], [Sb_2_*X*_8_]^2−^ [19], [Sb_2_*X*_9_]^3−^ [20,21], [Sb_2_*X*_10_]^4−^ [22], and [Sb_2_*X*_11_]^5−^ [23]. Low-dimensional Sb–OIMHs are generally classified as two-dimensional (2D), one-dimensional (1D), and zero-dimensional (0D) based on the connectivity modes of their inorganic anions [24,25]. Among these, 0D structures with isolated [Sb*X*_5_]^2−^ units typically exhibit efficient broadband emission due to self-trapped excitons (STEs) generated by exciton–lattice interactions and significant structural rearrangement in the excited state [26], making mononuclear [Sb*X*_5_]^2−^ units particularly common in the construction of photoluminescent (PL) 0D-OIMHs. It is worth noting that many OIMH systems containing [SbCl_5_]^2−^ exhibit high photoluminescence quantum yields (PLQY > 50%) due to their STE emission mechanism. This remarkable property has garnered significant attention in the research community [27,28].

Imidazolium-based ionic liquids offer exceptional structural diversity, enabling targeted adjustments and modifications [29,30]. This facilitates the “designability” of both the structure and properties of the resulting compounds. For example, our team assembled two distinct antimony-based luminescent OIMHs, namely, [Bzmim]_3_SbCl_6_ (reaction ratio 3:1) and [Bzmim]_2_SbCl_5_ (reaction ratio 2:1), by varying the synthesis ratio of the imidazolium ionic liquid [Bzmim]Cl (Bzmim = 1-benzyl-3-methylimidazolium) to Sb^3+^. Owing to distinct anionic configurations, [Bzmim]_3_SbCl_6_ exhibits green emission (*λ*_ex_ = 342 nm), whereas [Bzmim]_2_SbCl_5_ shows dual-band emission with blue (*λ*_ex_ = 310 nm) and red (*λ*_ex_ = 396 nm) components [31]. Furthermore, employing imidazolium ionic liquids with conformationally flexible butyl chains led to the isolation of *α*/*β*-[Bmmim]_2_SbCl_5_ isomers. These isomers possess different structural stacking patterns and exhibit varying degrees of anion structural distortion, resulting in unique luminescence characteristics [3]. While CCDC database analysis confirms that Sb(III)-based OIMHs featuring the mononuclear [SbCl_5_]^2−^ motif have been widely reported, studies specifically utilizing imidazolium-based ionic liquids to construct such compounds remain relatively scarce [2,13,14,15], and there is no detailed study of the structure–property relationship in this specific subset of materials.

**Figure 1:**
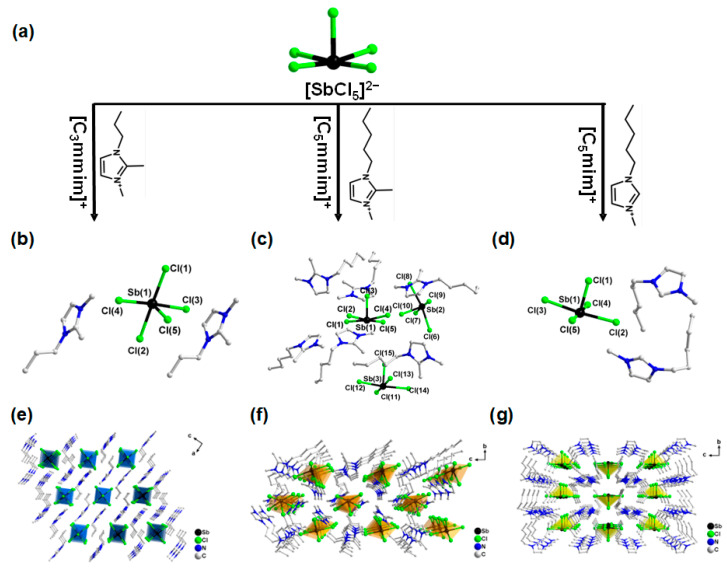
(**a**) Synthesis scheme for compounds **1**−**3**. (**b**) Asymmetric unit diagram of compound **1**. (**c**) Asymmetric unit diagram of compound **2**. (**d**) Asymmetric unit diagram of compound **3**. (**e**) View along the *b*-axis of the structural packing diagram of **1**. (**f**) View along the *a*-axis of the structural packing diagram of **2**. (**g**) View along the *a*-axis of the structural packing diagram of **3**. Hydrogen atoms are omitted for clarity.

Herein, three zero-dimensional Sb-based hybrid metal halides, [C_3_mmim]_2_SbCl_5_ (**1**, [C_3_mmim]^+^ = 1-propyl-2,3-dimethylimidazolium), [C_5_mmim]_2_SbCl_5_ (**2**, [C_5_mmim]^+^ = 1-pentyl-2,3-dimethylimidazolium), and [C_5_mim]_2_SbCl_5_ (**3**, [C_5_mim]^+^ = 1-pentyl-3-methylimidazolium), were synthesized by ionothermal methods (Figure 1a). Crucially, targeted structural modifications of the organic cations, specifically extending the alkyl chain length and strategically removing a methyl substituent, enable significant tuning of the PLQY within this [SbCl_5_]^2−^-based series. Achieving high PLQY, particularly in compound **3**, is essential as it directly enhances the material’s potential for practical applications, such as efficient X-ray scintillators and phosphors for white-light-emitting diodes (WLEDs). Structural analysis reveals that these modifications suppress non-radiative decay by increasing Sb–Sb distances to inhibit energy transfer and by optimizing the hydrogen bonding environment around the [SbCl_5_]^2−^ unit to minimize structural distortion. Furthermore, analysis of temperature-dependent photoluminescence indicates that the pronounced electron–phonon coupling promotes STE emission in all three compounds. The strength of this coupling, quantified by the Huang–Rhys factor (S), is a key determinant of non-radiative relaxation pathways; higher S values correlate strongly with increased phonon-assisted non-radiative losses. Thus, we successfully mitigate these losses through our structural design strategy. This work establishes clear structure–property relationships governing luminescence efficiency in 0D Sb–halide hybrids, providing a vital theoretical foundation for the rational design of high-performance luminescent materials.

## 2. Results and Discussion

### 2.1. Crystal Structure Description

Single-crystal X-ray diffraction (SCXRD) data collected at 100 or 150 K (crystallographic data are shown in Table S1) indicate that all three compounds exhibit zero-dimensional (0D) structural features. That is, discrete [SbCl_5_]^2−^ anions are separated by large-volume organic cations. Specifically, compound **1** ([C_3_mmim]_2_SbCl_5_) crystallizes in the monoclinic space group *P*2_1_/*c*, with its asymmetric unit containing one [SbCl_5_]^2−^ anion and two [C_5_mmim]^+^ cations (Figure 1b). The Sb–Cl bond lengths range from 2.376(3) to 2.753(9) Å (average 2.564 Å), and the Cl–Sb–Cl bond angles range from 84.93(9) to 92.37(10)° (average 88.25°). Compound **2** ([C_5_mmim]_2_SbCl_5_) belongs to the same *P*2_1_/*c* space group but has a more complex asymmetric unit, containing three [SbCl_5_]^2−^ anions and six [C_5_mmim]^+^ cations (Figure 1c). Its Sb–Cl bond lengths (2.372(11)−2.725(12) Å; average 2.567 Å) and bond angles ranges (83.79(4)−93.29(4)°; average 88.75°) are slightly broader than those of compound **1**. Compound **3** ([C_5_mim]_2_SbCl_5_) crystallizes in the orthorhombic space group *P*2_1_2_1_2_1_, with an asymmetric unit containing one [SbCl_5_]^2−^ anion and two [C_5_mim]^+^ cations (Figure 1d). The Sb–Cl bond length range is narrower (2.366(9)−2.626(12) Å; average 2.561 Å), and the bond angle range is more concentrated (87.12(4)−90.29(4)°; average 89.02°). As shown in Figure 1e–g, the cations and anions in the structure of compounds **1**–**3** are arranged alternately, forming a typical 0D structure. In addition, there are abundant hydrogen bonds in compounds **1**–**3** (Figures S1–S3), which play an important role in the construction and stability of the structure.

Powder X-ray diffraction (PXRD) analyses confirm the phase purity of compounds **1**–**3**, as experimental patterns align precisely with simulations derived from single-crystal X-ray diffraction (SCXRD) data (Figure S4a–c). Thermogravimetric analysis (TGA) reveals similar single-step decomposition profiles for all compounds, demonstrating thermal stability up to 280 °C (Figure S4d–f). This behavior is attributed to the inherent thermal stability and low volatility of the imidazolium-based ionic liquid cations, which effectively suppress premature structural decomposition.

**Figure 2:**
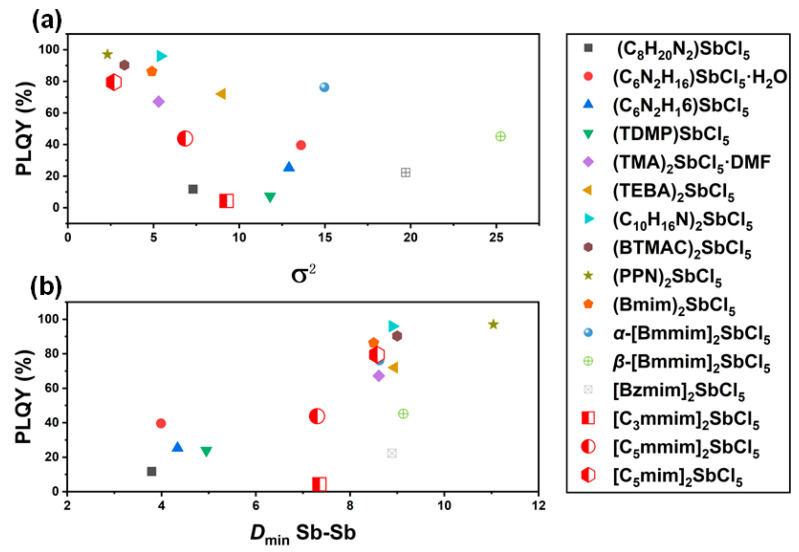
(**a**) Plot of the relationship between the *σ*^2^ and PLQY for the listed compounds. (**b**) Plot of the relationship between the shortest Sb–Sb distance (*D*_min_ Sb–Sb) and PLQY for the listed compounds.

To quantitatively evaluate how lattice distortions influence the PLQY of compounds **1**–**3**, we further employed two metrics: (1) bond angle variance (*σ^2^*) of *X*–*M*–*X* and (2) bond length distortion (Δ*d*) of *M*–*X*, calculated using established methods [32,33]:

(1) $$\sigma^{2}=\frac{1}{7}\sum_{n=1}^{8}{\left(\theta_{n}-90{}^{\circ}\right)}^{2}$$

(2) $$∆d=\frac{1}{5}\sum_{n=1}^{5}{\left[\frac{d_{n}-d}{d}\right]}^{2}$$

where *θ_n_* and *d_n_* are individual *X*–*M*–*X* bond angles and *M*–*X* bond lengths, respectively, and *d* represents their averaged values. For compounds **1**–**3**, the Δ*d* values (1.5 × 10^−4^, 1.7 × 10^−4^, and 1.5 × 10^−4^) show minimal variation, but the *σ^2^* values (9.3, 6.8, and 2.7) exhibit significant differences, with compound **1** exhibiting the most pronounced bond angle distortion. Additionally, a review of the existing literature on antimony chloride-based low-dimensional materials indicates that isolated units with smaller distortions (i.e., bond angle variance or bond length distortion) exhibit higher PLQY [34,35]. We systematically compiled recently reported hybrid metal halides containing [SbCl_5_]^2−^ units (Table 1) and investigated the correlation between their structural distortion and PLQY. We separately analyzed the relationship between bond angle distortion and PLQY for the listed compounds, as shown in Figure 2a. Bond angle distortion and PLQY exhibit a negative correlation trend, i.e., compounds with smaller structural distortions exhibit higher PLQY, which validates the reliability of previous studies. This is because emission centers with smaller distortions utilize lower electronic excitation energy during the structural reorganization process in the excited state. This minimizes non-radiative loss of electronic excitation energy, thereby enhancing stronger PLQY [36,37,38]. Clearly, this rule also applied to compounds **1**–**3**, with the degree of distortion in **3** being relatively lower than that in **1** and **2**, resulting in **3** having the highest PLQY.

To further investigate the origin of larger bond angle distortions, we compare and analyze the hydrogen bonding environment around the inorganic [SbCl_5_]^2−^ unit in compounds **1**–**3**. Structural analysis reveals significant differences in hydrogen bonding patterns among the compounds. Compounds **1** and **2** exhibit varying numbers of hydrogen bonds at the apical chlorine atoms of the pyramidal [SbCl_5_]^2−^ units (distinct from octahedral [SbCl_6_]^3−^ configurations); these interactions create an asymmetric hydrogen bonding environment along the central axis (Figure S5a,b) [44]. By contrast, compound **3** shows no such apical hydrogen bonding, resulting in a more symmetrical and balanced [SbCl_5_]^2−^ configuration (Figure S5c). The bond angle data show significant differences: the apical Cl–Sb–Cl bond angles in **1** and **2** have a wider range (**1**: 84.93°−88.18°; **2**: 85.34°−90.29°), while the bond angle range for **3** is significantly narrower (87.12°−89.31°), which is the key reason for the significant reduction in bond angle distortion. We speculate that the removal of the methyl group on the imidazole ring in **3** reduces the potential sites for hydrogen bonding with the terminal chlorine atoms of the [SbCl_5_]^2−^ unit. This allows the [SbCl_5_]^2−^ unit in **3** to form relatively balanced hydrogen bonding interactions along the central axis, effectively reducing its bond angle distortion and thereby helping to suppress non-radiative transitions.

In addition to the degree of distortion of [SbCl_5_]^2−^, the Sb–Sb distance is also an important factor affecting optical physical properties [21,45]. We analyze the [SbCl_5_]^2−^ anion configuration in compounds **1**–**3**, focusing on the Sb–Sb distance. As shown in Figure S5a–c, the Sb–Sb distances for the three compounds are illustrated. The shortest Sb–Sb distances for compounds **1**–**3** are 7.35, 7.30, and 8.57 Å, respectively. Since [SbCl_5_]^2−^ units based on imidazole groups are relatively rare in hybrid halides, we compiled the structural and photophysical properties of representative 0D hybrid metal halides containing [SbCl_5_]^2−^ units reported in recent years, revealing the relationship between the Sb–Sb distance and the PLQY (Table 1). Additionally, Figure 2b provides a more intuitive illustration of the relationship between the Sb–Sb distance and PLQY. It can be observed that larger Sb–Sb distances (>8.5 Å) exhibit higher PLQY, while distances below 8.5 Å exhibit lower PLQY. This can be attributed to the “concentration quenching” phenomenon, where shorter Sb–Sb distances can be regarded as showing high Sb concentrations, increasing energy transfer between Sb atoms. Conversely, longer Sb–Sb distances suppress energy transfer processes and then enhance the PLQY [46]. The distinct PLQY variations among compounds **1**–**3** stem from synergistic modulation of [SbCl_5_]^2−^ structural distortion and Sb–Sb distances through molecular engineering. Compared to compound **1**, the extended alkyl chains in compounds **2** and **3** increase imidazolium cation volume, expanding Sb–Sb distances and consequently suppressing inter-anion energy transfer to enhance PLQY. Furthermore, compound **3** achieves superior performance through elimination of the C2-methyl group present in compounds **1** and **2**, which decreases hydrogen bonding sites at the apical chlorine atoms. This modification establishes a more symmetrical hydrogen bonding environment along the [SbCl_5_]^2−^ central axis, minimizing structural distortion and bond angle deviation to effectively suppress non-radiative decay pathways, ultimately maximizing PLQY.

### 2.2. Optical Properties

**Figure 3:**
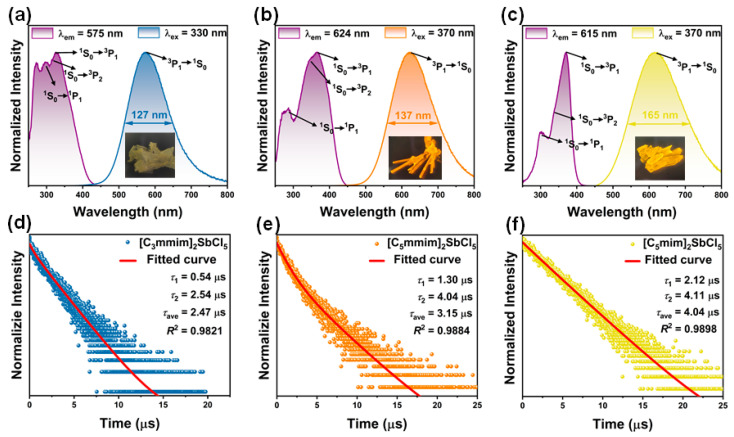
The steady-state PLE and PL spectra of [C_3_mmim]_2_SbCl_5_ (**a**), [C_5_mmim]_2_SbCl_5_ (**b**), and [C_5_mim]_2_SbCl_5_ (**c**) at room temperature. The time-resolved fluorescence spectra of [C_3_mmim]_2_SbCl_5_ (**d**), [C_5_mmim]_2_SbCl_5_ (**e**), and [C_5_mim]_2_SbCl_5_ (**f**) at 575, 624, and 615 nm, respectively. The fluorescence lifetimes were fitted, calculated, and labeled.

Solid-state absorption and fluorescence spectroscopy characterized the photophysical properties of these compounds. Based on the electronic transition involved in the ns^2^ ions [47,48], the UV absorption spectra (Figure S7a–c) reveal three peaks for each compound, assigned to Sb^3+^ transitions: **1** (300 nm: ^1^S_0_→^1^P_1_; 320 nm: ^1^S_0_→^3^P_2_; 340 nm: ^1^S_0_→^3^P_1_), **2** (300 nm: ^1^S_0_→^1^P_1_; 350 nm: ^1^S_0_→^3^P_2_; 370 nm: 1S_0_→3P_1_), and 3 (303 nm: ^1^S_0_→^1^P_1_; 330 nm: ^1^S_0_→^3^P_2_; 370 nm: ^1^S_0_→^3^P_1_). The steady-state photoluminescence excitation (PLE) and PL spectra of the compounds are shown in Figure 3a–c. Under 365 nm UV lamp irradiation, Crystal **1** emits yellow light, whereas Crystals **2** and **3** display orange and orange–yellow emission, respectively (insets, Figure 3a–c). At their optimal excitation wavelengths (*λ*_ex_ = 330 nm for **1**; 370 nm for **2**/**3**), all crystals exhibit broad emission bands, centering at *λ*_em_ = 575 nm for **1**, 624 nm for **2**, and 615 nm for **3**, respectively. Analysis of PL/PLE spectra reveals Stokes shifts of 245 nm for **1**, 254 nm for **2**, and 245 nm for **3**, with corresponding full width at half maxima (FWHM) values of 127 nm, 137 nm, and 165 nm, respectively. The measured PLQYs are 4.3% for **1**, 43.8% for **2**, and 79.5% for **3**, respectively. The Commission International del’Eclairage (CIE) chromaticity coordinates correspond to (0.4566, 0.5007), (0.5632, 0.4286), and (0.5240, 0.4578), respectively (Figure S8a–c) [27]. Time-resolved fluorescence decay curves were used to determine the fluorescence lifetimes of compounds **1**–**3**, as shown in Figure 3d–f. The lifetimes could be well fitted by a double exponential function (Equation (3)) [49]:

(3) $$I=I_{0}+A_{1}exp(-t/\tau_{1})+A_{2}exp(-t/\tau_{2})$$

The average lifetime can be calculated and obtained by the following Equation (4) [50]:

(4) $$\tau_{ave}=(A_{1}{\tau_{1}}^{2}+A_{1}{\tau_{1}}^{2})/(A_{1}\tau_{1}+A_{1}\tau_{2})$$

The fitting results indicate that the decay times of compounds **1**–**3** are 2.47, 3.15, and 4.05 microseconds, respectively. The specific parameters of the fitting process are detailed in Table S5. The broad emission bands, large Stokes shift, and long lifetime of PL suggest that the PL emission of **1**–**3** should originate from the ^3^P_1_→^1^S_0_ transition. In addition, we systematically recorded the photoluminescence spectra of compounds **1**–**3** at different excitation wavelengths. As shown in Figure S9a–c, although the luminescence intensity varies with the excitation wavelength, the luminescence peaks of all compounds remain almost unchanged (**1**: 575 nm; **2**: 624 nm; **3**: 615 nm). This excitation-independent behavior clearly confirms that the light source originates from the isolated ^3^P_1_→ ^1^S_0_ transition in the [SbCl_5_]^2−^ pyramid unit [27].

**Figure 4:**
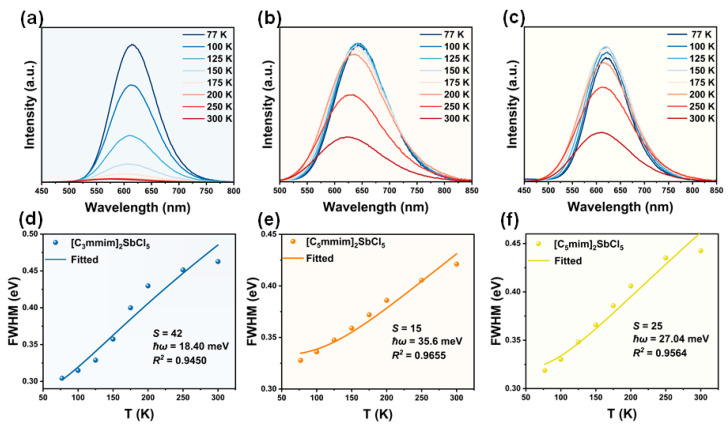
The temperature-dependent PL spectra and fitted physical parameters. (**a**–**c**) The PL spectra of compounds **1**–**3** at 77–300 K. (**d**–**f**) The relationship between the full width at half maximum (FWHM) and temperature (T) for compounds **1**–**3**.

By analyzing the temperature-dependent PL spectra (77–300 K) of compounds **1**–**3** and the fitted physical parameters, we further reveal the intrinsic mechanism underlying their PL performance differences. Temperature-dependent photoluminescence spectroscopy (77–300 K) reveals that compounds **1**–**3** exhibit pronounced thermal quenching (Figure 4a). Compound **1** exhibits severe thermal quenching, with its PL intensity decreasing to 2% of the 77 K value at 300 K. By contrast, compounds **2** and **3** are relatively stable in the 77–250 K range but undergo abrupt quenching above 250 K, retaining 38% and 53% of the 77 K baseline intensity at 300 K, respectively (Figure 4b,c). This reduction in PL intensity is attributed to enhanced thermal vibrations at high temperatures, which increase the lattice relaxation of the [SbCl_5_]^2−^ luminescent centers, thereby suppressing exciton recombination and enhancing non-radiative transitions [51]. Additionally, as the temperature increases, the PL positions exhibit varying degrees of blue shift (40, 25, and 16 nm for **1**–**3**, respectively), which can be clearly observed through temperature-dependent CIE coordinates (Figure S10a–f). These blue shifts may be attributed to lattice expansion leading to a decrease in crystal field strength [52]. As is well known, 0D hybrid halide structures exhibit soft lattices and strong electron–phonon coupling under excitation, thereby exhibiting broad PL spectrum with large Stokes shifts [27,53]. The soft lattice and electron–phonon coupling can be roughly evaluated by the Huang–Rhys (*S*) factor, which could be calculated through fitting the temperature-dependent FWHM curve according to the following Equation (5) [17,54]:

(5) $$FWHM=2.36\sqrt{S}\hbar \omega \sqrt{coth\left(\frac{\hbar \omega }{2kT}\right)}$$

where FWHM denotes the full width of half peak, *ħ* is Planck’s constant, *ω* is the phonon frequency, *k* is Boltzmann’s constant, and *T* is the temperature. Previous studies have shown that compounds with *S* ≥ 5 exhibit soft lattices and strong coupling between electrons and phonons [55]. In this work, the *S* values obtained by fitting for compounds **1**, **2**, and **3** are 42, 15, and 25, respectively (Figure 4d–f). Compounds **1**–**3** all exhibit strong electron–phonon interactions, which are conducive to the formation of STEs. Notably, **1** exhibits an exceptionally large *S* value, suggesting that **1** may exhibit intense lattice vibrations, which could enhance phonon-assisted non-radiative transitions, thereby impairing STE emission efficiency [56].

### 2.3. Density Functional Theory (DFT) Calculations 

**Figure 5:**
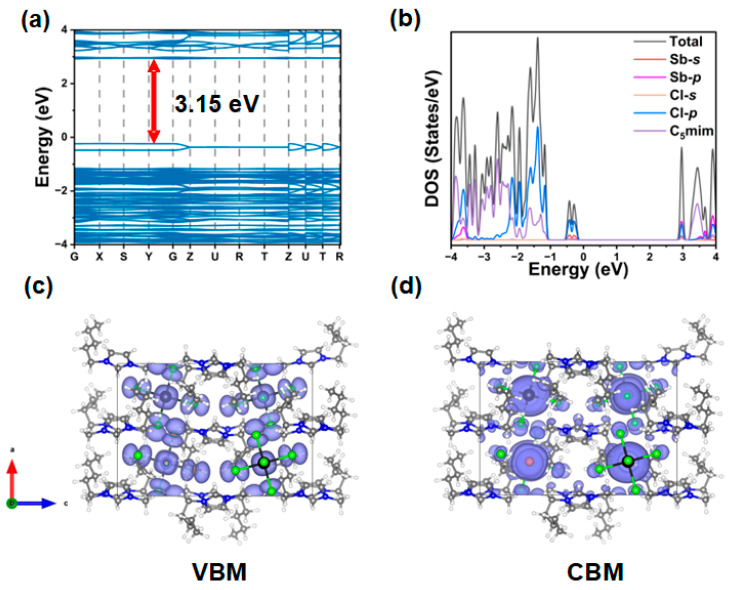
DFT calculations of **3**. (**a**) Electronic energy band structures of compound **3**. (**b**) Orbital-resolved DOS calculations for **3**. The charge density distribution of VBM (**c**) and CBM (**d**) of **3**. The single cell of **3** was optimized according to crystallographic symmetry. Black sphere, Sb; green sphere, Cl; blue sphere, N; dark grey sphere, C; light grey sphere, H.

The electronic properties of compound **3** are calculated theoretically. The calculated band structure shows a direct bandgap of 3.15 eV (Figure 5a). For direct bandgap compounds, the absorption and excitation processes of light do not require additional phonon assistance. The recombination process in direct bandgap compounds is conducive to the formation of radiative pathways. Notably, the electronic structure of the valence band maximum (VBM) and conduction band minimum (CBM) is nearly flat, and the flatness of the two bands indicates a strong quantum confinement effect [57]. Figure 5b shows the orbital-resolved density of states (DOS) for compound **3**, where the VBM is primarily composed of Sb-5s and Cl-3p, while the CBM is primarily composed of Sb-5p and Cl-3p. The partial charge density curves for the VBM and CBM are shown in Figure 5c,d. These results confirm that the broadband emission of **3** mainly originates from isolated inorganic [SbCl_5_]^2−^ pyramids, which is consistent with reports on antimony-based hybrid metal halides [58].

## 3. Materials and Methods

**Reagents:** Antimony(III) chloride (SbCl_3_, 99%) was purchased from Adamas Reagent Co, Ltd. (Jiaxing, China). Ionic liquids including 1-propyl-2,3-dimethylimidazolium chloride ([C_3_mmim]Cl, 99%), 1-pentyl-2,3-dimethylimidazolium chloride ([C_5_mmim]Cl, 99%), and 1-pentyl-3-methylimidazolium chloride ([C_5_mim]Cl, 99%) were purchased from Lanzhou GreenChem ILs, LICP, CAS (Lanzhou, China); all reagents were used without further purification.

**Synthesis of 1:** Amounts of 0.2307 g of SbCl_3_ and 0.3626 g of [C_3_mmim]Cl (molar ratio about 1:2) were placed into a 20 mL sealed polytetrafluoroethylene hot press reactor, heated in an oven at 120 °C for 6 h, and then naturally cooled to room temperature to obtain transparent colorless block-like crystals with a yield of 99%. EA: Calcd (%): C: 33.28, H: 5.24, N: 9.70. Found (%): C: 33.30, H: 6.04, N: 9.71.

**Synthesis of 2:** Amounts of 0.2383 g SbCl_3_ and 0.4445 g [C_5_mimm]Cl (molar ratio of about 1:2) were put into a 28 mL sealed polytetrafluoroethylene thermocompression kettle reactor without iron shell, heated in an oven at 120 °C for 3 h, and then naturally cooled to room temperature to obtain transparent colorless prismatic crystals with a yield of 93%. EA: Calcd (%): C: 37.91, H:6.05, N: 8.84. Found (%): C: 37.42, H: 6.13, N: 8.76.

**Synthesis of 3:** Amounts of 0.4586 g of SbCl_3_ and 1.0026 g of [C_5_mim]Cl (molar ratio about 2:4) were put into a 20 mL sealed polytetrafluoroethylene thermocompression kettle reactor without iron shell, heated in an oven at 140 °C for 6 h, and naturally cooled to room temperature to obtain colorless transparent bulk-like crystals in a yield of 77%. EA: Calcd (%): C: 35.70, H: 5.66, N: 9.25. Found (%): C: 34.82, H: 5.60, N: 9.00.

**Single-crystal X-ray diffraction (SCXRD):** The crystal data for compound **1** were recorded on a Bruker APEX-II (MoKα, λ = 0.71073 Å) single-crystal diffractometer manufactured by Bruker Corporation, Ettlingen, Germany, at a temperature of 150 K. The crystal data for Compound **2** were recorded using a Rigaku XtaL AB Synergy R microfocus (MoKα, λ = 0.71073 Å) single-crystal diffractometer manufactured by Rigaku Corporation, Tokyo, Japan, at a temperature of 100 K. The crystal data for compound **3** were recorded using a Supernova E dual microfocus (MoKα, λ = 0.71073 Å) single-crystal X-ray diffractometer produced by Agilent Technologies in the UK at a temperature of 100 K. The structures were solved by direct methods and refined by full-matrix least squares on *F*^2^ using the SHELX-2018 program package [59]. All non-hydrogen atoms were refined anisotropic ally, while the hydrogen atoms attached to the C atoms were refined anisotropic ally. All non-hydrogen atoms were refined by anisotropy, while the hydrogen atoms attached to the C atoms were located in geometrically calculated positions. The empirical formulas were verified by EA. Table S1 lists the crystallographic data and structural refinement details for compounds **1**, **2**, and **3**. Partial bond lengths and angles are listed in Tables S2 and S3, respectively. Table S4 lists the detailed hydrogen bonding data of the compounds. [CCDC NO. 2405568, 2405570, and 2405571 contain the supplementary crystallographic data for this paper. These data can be obtained free of charge from the Cambridge Crystallographic Data Centre via www.ccdc.cam.ac.uk/data_request/cif, accessed on 3 July 2025.]

**Powder X-ray diffraction (PXRD):** Powder X-ray diffraction (PXRD) patterns of compounds **1**–**3** were collected at room temperature using a Rigaku MiniFlex II diffractometer (CuKα radiation, λ = 1.54178 Å) manufactured by Rigaku Americas Corporation (Woodlands, TX, USA). The samples were finely ground, loaded onto a silicon zero-background holder, and flattened for uniform irradiation. Data collection parameters were as follows: 2*θ* range = 5–40°, scan rate = 1°/min, operating voltage = 30 kV and 15 mA.

**Thermogravimetric analyses (TG):** TGA was performed on a NETZSCH STA449F3 instrument manufactured by NETZSCH, Selb, Germany, in a N_2_ atmosphere. The sample (approximately 15 mg) was heated from 20 °C to 800 °C at a heating rate of 10 °C min^−1^.

**Solid-state UV–visible absorption spectroscopy (UV-vis):** Solid-state diffuse reflectance data in the 800–200 nm range was recorded at room temperature (RT) using a Shimadzu 2600 UV-vis spectrophotometer manufactured by Shimadzu Corporation in Shanghai, China. A BaSO_4_ integration sphere was calibrated as the 100% reflectance reference with 5 nm slit width prior to sample measurement. Absorption data were then obtained from the reflectance spectra using the Kubelka–Munk function α/*S* = (1 − *R*)^2^/2*R*, where α denotes the absorption coefficient, *S* denotes the scattering coefficient, and *R* denotes the reflectance.

**Steady-state photoluminescence spectra:** Steady-state photoluminescence spectroscopy: Photoluminescence excitation (PLE), photoluminescence (PL) spectra, and photoluminescence quantum yield (PLQY) were measured using an FLS1000 UV/Vis/NIR fluorescence spectrometer manufactured by Edinburgh Scientific in the Edinburgh, UK. During testing, the PL spectrum was measured using a 450W xenon lamp as the excitation source, with the slit width set to 0.1. The PLQY test utilized a 30 mW, 375 nm laser from Horiba Jobin Yvon in Palaiseau, France.

**Time-resolved photoluminescence spectra:** We measured time-resolved PL spectra on an FLS1000 UV/V/NIR fluorescence spectrometer from Edinburgh Instruments in the Livingston, UK.

**Temperature-dependent photoluminescence spectra:** Temperature-dependent PL spectra were measured on an FLS980 fluorescence spectrometer at Edinburgh Instruments in the UK, with a temperature range of 77–320 K. During the test process, the heating rate was set to 30 K/min, and each spectrum was recorded after stabilizing at each temperature value for three minutes.

**DFT calculations:** According to the single-crystal structure refinement results, DFT calculations of [C_5_mimm]SbCl_5_ were implemented in the Vienna ab initio simulation package (VASP 6.3.2) [60,61]. The generalized gradient approximation (GGA) for the exchange-correlation term with the Perdew–Burke–Ernzerhof (PBE) exchange-correlation functional was applied for electron–electron exchange-correlation processes. Projected augmented wave (PAW) potentials were used with the valence states 2s and 2p for C and N, 5s and 5p for Sb, and 3s and 3p for Cl. To ensure sufficient accuracy, a cut-off energy of 500 eV for the plane wave expansion was chosen, self-consistent field (SCF) computations were set to a convergence criterion of 1 × 10^−5^ eV, and the force criterion was 0.02 eV Å^−1^. The DFT calculations in this study were carried out on the Big Earth Date Cloud Service Platform.

## 4. Conclusions

This work systematically reveals the structure–property relationship of 0D antimony-based hybrid halides constructed from imidazole-based ionic liquids. Three compounds (**1**–**3**) are synthesized by ionothermal method. It is found that compounds **2** and **3**, which have longer alkyl chains in the cation, increase the Sb–Sb distance, effectively isolating the [SbCl_5_]^2−^ units and suppressing energy transfer, thereby significantly enhancing luminescence intensity. Furthermore, the methyl reduction modification of the imidazole ring in compound **3** effectively reduces the structural distortion of the [SbCl_5_]^2−^ unit, minimizing non-radiative transition pathways, resulting in superior luminescence performance of compound **3** compared to compound **2**. Finally, analysis of the temperature-dependent PL spectra revealed significant electron–phonon coupling in compounds **1**–**3**, which effectively promotes the formation of STEs. However, compound **1** exhibits an extremely high *S*, which significantly enhances phonon-mediated non-radiative decay and ultimately reduces its emission efficiency. This work deepens the understanding of the structure–property relationship between structural regulation strategies of the [SbCl_5_]^2−^ unit and its photophysical properties, providing a theoretical basis for designing high-performance luminescent materials.

## Figures and Tables

**Table 1:** Summary of structural parameters and photophysical properties of 0D OIMHs based on [SbCl_5_]^2−^.

Compound	*D*_min_ Sb–Sb (Å)	*d_n_* (10^−4^)	*θ_n_*	PLQY (%)	Ref.
(C_8_H_20_N_2_)SbCl_5_	3.79	5.7	7.3	11.8	[39]
(C_6_N_2_H_16_)SbCl_5_·H_2_O	3.99	9.1	13.6	39.6	[14]
(C_6_N_2_H_16_)SbCl_5_	4.34	4.8	12.9	25.3	[14]
(TDMP)SbCl_5_	4.95	8.3	11.8	24.0	[40]
(TMA)_2_SbCl_5_·DMF	8.61	1.5	5.3	67.2	[41]
(TEBA)_2_SbCl_5_	8.94	1.6	9.0	72.0	[13]
(C_10_H_16_N)_2_SbCl_5_	8.90	1.5	5.4	96.0	[39]
(BTMAC)_2_SbCl_5_	9.00	1.4	3.3	90.3	[42]
(PPN)_2_SbCl_5_	11.04	13.7	2.3	97.0	[2]
(Bmim)_2_SbCl_5_	8.50	3.3	4.9	86.3	[43]
*α*-[Bmmim]_2_SbCl_5_	8.62	2.3	15.0	76.2	[3]
*β*-[Bmmim]_2_SbCl_5_	9.13	2.7	25.3	45.2	[3]
[Bzmim]_2_SbCl_5_	8.89	4.9	19.7	22.3	[31]
[C_3_mmim]_2_SbCl_5_	7.35	1.5	9.3	4.3	This work
[C_5_mmim]_2_SbCl_5_	7.30	1.3	6.8	43.8	This work
[C_5_mim]_2_SbCl_5_	8.57	1.5	2.7	79.5	This work

## Data Availability

The original contributions presented in this study are included in the article/Supplementary Materials. Further inquiries can be directed to the corresponding author.

## Supplementary Materials

The following supporting information can be downloaded at: https://www.mdpi.com/article/10.3390/molecules30163431/s1, Table S1: Crystallographic data and structural refinement details for title compounds; Table S2: Selected bond lengths (Å) for compounds **1**, **2**, and **3**; Table S3: Selected bond angles (°) for compounds **1**, **2**, and **3**; Table S4: Hydrogen bonds for compounds **1**, **2**, and **3**; Table S5: Summary of relevant parameters in the fluorescence lifetime fitting process; Figure S1: Supramolecular structure of compound **1** observed from *a* (**a**) and *b* (**b**) axes, respectively; Figure S2: Supramolecular structure of compound **2** observed from *a* (**a**) and *b* (**b**) axes, respectively; Figure S3: Supramolecular structure of compound **3** observed from *a* (**a**) and *b* (**b**) axes, respectively; Figure S4: Experimental and simulated PXRD patterns of compounds **1** (**a**), **2** (**b**), and **3** (**c**). TG curves of compounds **1** (**d**), **2** (**e**), and **3** (**f**); Figure S5: The hydrogen bonds between inorganic [SbCl_5_]^2−^ units and organic cations of **1**–**3** compounds (**a**–**c**). The red numbers represent the number of hydrogen bonds around the Cl ions; Figure S6: Diagrams showing the Sb–Sb distances between [SbCl_5_]^2−^ units in compounds **1** (**a**), **2** (**b**), and **3** (**c**); Figure S7: The UV-vis optical absorption spectra of [C_3_mmim]_2_SbCl_5_ (**a**), [C_5_mmim]_2_SbCl_5_ (**b**), and [C_5_mim]_2_SbCl_5_ (**c**); Figure S8: CIE coordinate diagrams for compounds **1** (**a**), **2** (**b**), and **3** (**c**); Figure S9: Emission spectra of compounds **1** (**a**), **2** (**b**), and **3** (**c**) under different excitations; Figure S10: Normalized variable temperature spectra of compounds **1** (**a**), **2** (**b**), and **3** (**c**). Variable temperature CIE coordinate diagrams of compounds **1** (**d**), **2** (**e**), and **3** (**f**).
