# Supplementary material for: Tuning the Structure and Photoluminescence of [SbCl5]2−-Based Halides via Modification of Imidazolium-Based Cations
Source: Molecules. 2025 Aug 20;30(16):3431. doi: 10.3390/molecules30163431 (PMC12388840; doi:10.3390/molecules30163431)
Supplement: Supplementary file 1 [file molecules-30-03431-s001.zip › molecules-3769115-supplementary.pdf]

Supporting Information for

Tuning the Structure and Photoluminescence of $[SbCl_5]^{2-}$-Based Halides via Modification of Imidazolium-Based Cations

Guoyang Chen[1,2,3], Xinping Guo[1,2,3], Haowei Lin[2,3], Zhizhuan Zhang[2,3], Abdusalam Ablez [1,2,3], Yuwei Ren[1,2,3], Kezhao Du[4,*], Xiaoying Huang[2,3,*]

[1] College of Chemistry, Fuzhou University, Fuzhou 350108, China; chenguoyang@fjirsm.ac.cn (G.C.); guoxinping@fjirsm.ac.cn (X.G.); abdslm@fjirsm.ac.cn (A.A.); renyuwei@fjirsm.ac.cn (Y.R.).

[2] State Key Laboratory of Structural Chemistry, Fujian Institute of Research on the Structure of Matter, Chinese Academy of Sciences, Fuzhou 350002, China; linhw@fjirsm.ac.cn (H.L.); zhangzhizhuan@stu.scu.edu.cn (Z.Z.).

[3] Fujian College, University of Chinese Academy of Sciences, Fuzhou 100049, China.

[4] Fujian Provincial Key Laboratory of Advanced Materials Oriented Chemical Engineering, Fujian Normal University, Fuzhou 350007, China.

Correspondence: duke@fjnu.edu.cn (K.D.); xyhuang@fjirsm.ac.cn (X.H.).

Table S1. Crystal data and structure refinement for compounds **1**, **2**, and **3** at 150 K or 100 K

**Identification code**	**1**	**2**	**3**
CCDC number	2405568	2405570	2405571
Empirical formula	$C_{16}H_{30}N_4SbCl_5$	$C_{20}H_{38}N_4SbCl_5$	$C_{18}H_{34}N_4SbCl_5$
Formula weight	577.44	633.54	605.49
Temperature/K	150.00	100.00	100.00
Crystal system	monoclinic	monoclinic	orthorhombic
Space group	$P2_1/c$	$P2_1/c$	$P2_12_12_1$
a/Å	10.493(6)	12.8979(2)	11.9189(7)
b/Å	7.358(4)	64.3419(10)	12.3231(7)
c/Å	30.618(16)	10.2445(2)	17.5937(11)
α/°	90	90	90
β/°	91.695(7)	92.252(10)	90
γ/°	90	90	90
Volume/Å^3	2363(2)	8495.1(2)	2584.1(3)
Z	4	12	4
ρ_{calc}g/cm^3	1.632	1.486	1.556
μ/mm^{-1}	1.742	1.461	1.597
F(000)	1160.0	3864.0	1224.0
Crystal size/mm^3	0.20*0.20*0.15	0.25*0.20*0.15	0.70*0.20*0.15
Radiation	Mo$K\alpha$ (λ = 0.71073)	Mo$K\alpha$ (λ = 0.71073)	Mo$K\alpha$ (λ = 0.71073)
2θ range for data collection/°	3.884 to 50.1	3.222 to 61.594	4.036 to 60.54
Index ranges	$-12 \le h \le 12$, $-7 \le k \le 8$, $-23 \le l \le 36$	$-16 \le h \le 18$, $-86 \le k \le 85$, $-12 \le l \le 10$	$-13 \le h \le 16$, $-17 \le k \le 16$, $-24 \le l \le 23$
Reflections collected	8966	97988	18185
Independent reflections	4092[R_{int} =0.0221, R_{sigma} = 0.0335]	21153[R_{int} = 0.0523, R_{sigma} = 0.0484]	6777[R_{int} =0.0351, R_{sigma} = 0.0478]
Data/restraints/parameters	4092/744/335	21153/40/849	6777/0/258
Goodness-of-fit on F^2	1.018	1.209	1.009
Final R indexes [I>=2σ(I)]	R_1 = 0.0610, wR_2 = 0.1493	R_1 = 0.0535, wR_2 = 0.1063	R_1 = 0.0335, wR_2 = 0.0602
Final R indexes [all data]	R_1 = 0.0696, wR_2 = 0.1554	R_1 = 0.0667, wR_2 = 0.1095	R_1 = 0.0555, wR_2 = 0.0717
Largest diff. peak/hole / e Å^{-3}	1.53/-0.86	1.04/-0.85	0.57/-1.12

[a] $wR_1 = \sum \| F_o | - | F_c \| / \sum | Fo |$, [b] $wR_2 = [\sum w(F_o^2 - F_c^2)^2 / \sum w(F_o^2)^2]^{1/2}$

Table S2. Selected bond lengths (Å) for compounds **1**, **2**, and **3**

	**1**		
Sb(1)-Cl(5)	2.376(3)	Sb(1B)-Cl(5B)	2.26(10)
Sb(1)-Cl(1)	2.624(3)	Sb(1B)-Cl(1)	2.621(10)
Sb(1)-Cl(2)	2.616(3)	Sb(1B)-Cl(2)	2.668(9)
Sb(1)-Cl(3)	2.590(3)	Sb(1B)-Cl(3)	2.753(9)
Sb(1)-Cl(4)	2.644(3)	Sb(1B)-Cl(4)	2.487(9)

	**2**		
Sb(1)-Cl(1)	2.5874(12)	Sb(2)-Cl(9)	2.5588(13)
Sb(1)-Cl(2)	2.6238(11)	Sb(2)-Cl(10)	2.3912(11)
Sb(1)-Cl(2)	2.3776(11)	Sb(3)-Cl(11)	2.5663(12)
Sb(1)-Cl(4)	2.6075(12)	Sb(3)-Cl(12)	2.5521(12)
Sb(1)-Cl(5)	2.6027(11)	Sb(3)-Cl(13)	2.6363(12)
Sb(2)-Cl(6)	2.5861(13)	Sb(3)-Cl(14)	2.7257(12)
Sb(2)-Cl(7)	2.6291(13)	Sb(3)-Cl(15)	2.3721(11)
Sb(2)-Cl(8)	2.6688(13)		

**3**	
Sb(1)-Cl(1)	2.3668(9)
Sb(1)-Cl(2)	2.6263(12)
Sb(1)-Cl(2)	2.6202(12)
Sb(1)-Cl(4)	2.6145(11)
Sb(1)-Cl(5)	2.5786(11)

Table S3. Selected bond angles (°) for compounds **1**, **2**, and **3**

	**1**		
Cl(5)-Sb(1)-Cl(1)	88.18(11)	Cl(5B)-Sb(1B)-Cl(1)	85.1(5)
Cl(5)-Sb(1)-Cl(2)	87.52(10)	Cl(5B)-Sb(1B)-Cl(2)	79.5(5)
Cl(5)-Sb(1)-Cl(3)	84.93(9)	Cl(5B)-Sb(1B)-Cl(3)	80.9(5)
Cl(5)-Sb(1)-Cl(4)	86.01(10)	Cl(5B)-Sb(1B)-Cl(4)	88.6(5)
Cl(1)-Sb(1)-Cl(4)	90.69(10)	Cl(1)-Sb(1B)-Cl(2)	163.9(5)
Cl(2)-Sb(1)-Cl(1)	175.46(11)	Cl(1)-Sb(1B)-Cl(3)	85.4(3)
Cl(2)-Sb(1)-Cl(4)	87.57(9)	Cl(2)-Sb(1B)-Cl(3)	87.7(3)
Cl(3)-Sb(1)-Cl(1)	88.69(9)	Cl(4)-Sb(1B)-Cl(1)	94.4(3)
Cl(3)-Sb(1)-Cl(2)	92.37(10)	Cl(4)-Sb(1B)-Cl(2)	89.8(3)
Cl(3)-Sb(1)-Cl(4)	170.93(10)	Cl(4)-Sb(1B)-Cl(3)	169.4(5)

	**2**		
Cl(1)-Sb(1)-Cl(2)	88.64(4)	Cl(9)-Sb(2)-Cl(8)	89.96(4)
Cl(1)-Sb(1)-Cl(4)	172.94(4)	Cl(10)-Sb(2)-Cl(6)	85.36(4)
Cl(1)-Sb(1)-Cl(5)	89.18(4)	Cl(10)-Sb(2)-Cl(7)	90.06(4)
Cl(3)-Sb(1)-Cl(1)	87.65(4)	Cl(10)-Sb(2)-Cl(8)	83.79(4)
Cl(3)-Sb(1)-Cl(2)	88.57(4)	Cl(10)-Sb(2)-Cl(9)	90.29(5)
Cl(3)-Sb(1)-Cl(4)	85.34(4)	Cl(11)-Sb(3)-Cl(13)	176.66(4)
Cl(3)-Sb(1)-Cl(5)	86.38(4)	Cl(11)-Sb(3)-Cl(14)	91.79(4)
Cl(4)-Sb(1)-Cl(2)	90.37(4)	Cl(12)-Sb(3)-Cl(11)	89.53(4)
Cl(5)-Sb(1)-Cl(2)	174.58(4)	Cl(12)-Sb(3)-Cl(13)	90.75(4)
Cl(5)-Sb(2)-Cl(4)	91.21(4)	Cl(12)-Sb(3)-Cl(14)	174.49(4)
Cl(6)-Sb(2)-Cl(7)	87.82(5)	Cl(13)-Sb(3)-Cl(14)	87.62(4)
Cl(6)-Sb(2)-Cl(8)	169.09(4)	Cl(15)-Sb(3)-Cl(11)	88.39(4)
Cl(7)-Sb(2)-Cl(8)	93.29(4)	Cl(15)-Sb(3)-Cl(12)	87.50(3)
Cl(9)-Sb(2)-Cl(6)	88.99(5)	Cl(15)-Sb(3)-Cl(13)	88.30(4)

	**3**		
Cl(1)-Sb(1)-Cl(2)	87.12(4)	Cl(4)-Sb(1)-Cl(2)	90.28(4)
Cl(1)-Sb(1)-Cl(3)	87.12(4)	Cl(4)-Sb(1)-Cl(3)	89.90(4)
Cl(1)-Sb(1)-Cl(4)	88.81(4)	Cl(5)-Sb(1)-Cl(2)	89.33(4)

Cl(1)-Sb(1)-Cl(5)	89.31(4)	Cl(5)-Sb(1)-Cl(3)	90.29(4)
Cl(3)-Sb(1)-Cl(2)	174.28(4)	Cl(5)-Sb(1)-Cl(4)	178.10(4)

Table S4. Hydrogen bonds for compounds **1**, **2**, and **3**

**1**				
D-H···A	*d*(D-H) (°)	*d*(H···A) (°)	*d*(D···A) (Å)	<(DHA) (°)
C(9)-H(9A)···Cl(3)#1	0.95	2.78	3.673(10)	157.3
C(10)-H(10A)···Cl(2)#2	0.95	2.81	3.652(10)	148.4
C(12)-H(12A)···Cl(5)#1	0.98	2.64	2.583(9)	162.0
C(12)-H(12B)···Cl(5B)#3	0.98	2.85	3.441(16)	119.3
C(12)-H(12B)···Cl(3)#3	0.98	2.85	3.709(9)	147.4
C(13)-H(13A)···Cl(2)#4	0.98	2.90	3.840(12)	162.1
C(14)-H(14A)···Cl(2)#4	0.99	2.88	3.816(8)	157.4
C(1)-H(1B)···Cl(4)#5	0.95	2.73	3.627(11)	158.1
C(2)-H(2A)···Cl(1)#6	0.95	2.78	3.637(13)	150.6
C(4)-H(4A)···Cl(5)#5	0.98	2.91	3.631(15)	131.0
C(5)-H(5B)···Cl(1)#7	0.98	2.89	3.850(13)	166.4
C(5)-H(5C)···Cl(4)#8	0.98	2.97	3.625(13)	124.9
C(6)-H(6B)···Cl(1)#6	0.99	2.87	3.800(14)	157.4
C(1B)-H(1BA)···Cl(2)	0.95	2.88	3.48(2)	122.0
C(1B)-H(1BA)···Cl(4)	0.95	2.78	3.659(19)	154.0
C(2B)-H(2BA)···Cl(1)#7	0.95	2.59	3.43(2)	147.9
C(6B)-H(6C)···Cl(1)#7	0.95	2.72	3.59(3)	147.2

Symmetry codes: #1 -*x*,1/2+*y*,1/2-*z*; #2 1-*x*,1/2+*y*,1/2-*z*; #3-*x*,-1/2+*y*,1/2-*z*; #4 1-*x*,-1/2+*y*,1/2-*z*; #5 +*x*,1+*y*,+*z*; #6 1 +*x*,1+*y*,+*z*; #7 1+*x*,+*y*,+*z*; #8 1 -*x*,1-*y*,-*z*

**2**				
D-H···A	*d*(D-H) (°)	*d*(H···A) (°)	*d*(D···A) (Å)	<(DHA) (°)
C(1)-H(1A)···Cl(2)#1	0.95	2.9	3.600(5)	131.9
C(1)-H(1A)···Cl(4)#1	0.95	2.82	3.633(5)	144.9
C(2)-H(2A)···Cl(14)#2	0.95	2.92	3.733(5)	143.9
C(4)-H(4A)···Cl(4)#1	0.98	2.82	3.749(5)	158.8
C(4)-H(4B)···Cl(15)	0.98	2.71	3.676(5)	170.2
C(4)-H(4C)···Cl(5)	0.98	2.79	3.723(5)	158.5
C(5)-H(5B)···Cl(5)	0.98	2.87	3.788(5)	155.9
C(5)-H(5C)···Cl(12)	0.98	2.86	3.678(5)	141.2
C(11)-H(11A)···Cl(5)	0.95	2.97	3.816(6)	149.8
C(12)-H(12A)···Cl(1)	0.95	2.74	3.566(5)	146
C(14)-H(14A)···Cl(1)	0.98	2.73	3.694(6)	168.1
C(14)-H(14C)···Cl(6)#2	0.98	2.77	3.729(5)	165
C(15)-H(15B)···Cl(6)#2	0.98	2.89	3.848(5)	165.2
C(15)-H(15C)···Cl(2)#1	0.98	2.89	3.831(5)	162

C(21)-H(21A)···Cl(7)#3	0.95	2.88	3.442(5)	118.8
C(21)-H(21A)···Cl(10)#3	0.95	2.76	3.635(5)	153.7
C(22)-H(22A)···Cl(4)	0.95	2.63	3.560(5)	166
C(24)-H(24A)···Cl(6)#3	0.98	2.81	3.782(6)	174
C(24)-H(24B)···Cl(2)#4	0.98	2.69	3.625(6)	158.9
C(24)-H(24C)···Cl(14)#3	0.98	2.92	3.872(6)	163.7
C(24)-H(24C)···Cl(15)#3	0.98	2.93	3.513(5)	119.2
C(25)-H(25B)···Cl(11)#3	0.98	2.75	3.707(5)	166.8
C(25)-H(25C)···Cl(2)#4	0.98	2.93	3.908(5)	173.8
C(26)-H(26A)···Cl(1)#3	0.99	2.68	3.534(6)	144.4
C(26)-H(26B)···Cl(5)	0.99	2.93	3.920(5)	174
C(32)-H(32A)···Cl(1)	0.95	2.75	3.586(5)	147.9
C(34)-H(34A)···Cl(13)#2	0.98	2.79	3.631(5)	144.4
C(34)-H(34A)···Cl(15)#2	0.98	2.86	3.659(5)	139.1
C(34)-H(34B)···Cl(10)#2	0.98	2.93	3.694(5)	135.9
C(34)-H(34C) ···Cl(5)#2	0.98	2.85	3.820(5)	171.1
C(35)-H(35B)···Cl(5)#2	0.98	2.79	3.716(5)	158.4
C(35)-H(35C)···Cl(9)2	0.98	2.54	3.483(5)	161.4
C(36)-H(36A)···Cl(3)	0.99	2.94	3.762(5)	141.5
C(36)-H(36B)···Cl(7)#5	0.99	2.78	3.622(5)	142.7
C(41)-H(41A)···Cl(1)	0.95	2.87	3.617(5)	136.9
C(41)-H(41A)···Cl(2)	0.95	2.96	3.782(5)	145.5
C(42)-H(42A)···Cl(14)#2	0.95	2.85	3.765(5)	161.2
C(44)-H(44C)···Cl(11)#5	0.98	2.9	3.735(5)	143.9
C(45)-H(45B)···Cl(12)#6	0.98	2.83	3.797(5)	169.1
C(45)-H(45C)···Cl(11)#5	0.98	2.82	3.468(5)	124.4
C(46)-H(46A)···Cl(13)#2	0.99	2.9	3.782(5)	149.3
C(51)-H(51A)···Cl(4)	0.95	2.86	3.668(5)	143.2
C(51)-H(51A)···Cl(5)	0.95	2.95	3.701(5)	136.7
C(52)-H(52A)···Cl(7)#3	0.95	2.77	3.461(6)	130.6
C(54)-H(54A)···Cl(5)	0.98	3	3.879(6)	150.3
C(55)-H(55B)···Cl(9)	0.98	2.75	3.398(6)	124.4
C(55)-H(55C)···Cl(8)#7	0.98	2.75	3.687(5)	160.7
C(56)-H(56A)···Cl(8)#3	0.99	2.92	3.872(6)	162.3
C(57)-H(57A)···Cl(7)#3	0.99	2.97	3.715(6)	132.4

Symmetry codes: #1 +*x*,+*y*,-1+*z*; #2 1+*x*,+*y*,+*z*; #3 +*x*,+*y*,1+*z*; #4 -1+*x*,+*y*,+*z*; #5 1 +*x*,+*y*,1+*z*; #6 1-*x*,1-*y*,1-*z*; #7 +*x*,1/2-*y*,1/2+*z*

**3**				
D-H···A	*d*(D-H) (°)	*d*(H···A) (°)	*d*(D···A) (Å)	<(DHA) (°)
C(1)-H(1A)···Cl(2)#1	0.95	2.76	3.560(5)	142.2
C(1)-H(1A)···Cl(5)#1	0.95	2.89	3.643(5)	136.9
C(2)-H(2A)···Cl(3)#2	0.95	2.68	3.562(5)	155.3
C(3)-H(3A)···Cl(2)	0.95	2.98	3.767(5)	141.3
C(3)-H(3A)···Cl(3)#3	0.95	2.87	3.650(5)	139.6

C(4)-H(4A)···Cl(5)#1	0.98	2.85	3.740(5)	151.9
C(4)-H(4B)···Cl(5)#3	0.98	2.97	3.706(5)	132.6
C(5)-H(5A)···Cl(4)#2	0.99	2.84	3.714(5)	147.8
C(5)-H(5B)···Cl(2)	0.99	2.72	3.671(5)	160.6
C(6)-H(6A)···Cl(4)	0.99	2.91	3.610(5)	128.8
C(10)-H(10A)···Cl(3)#4	0.95	2.76	3.569(5)	142.9
C(10)-H(10A)···Cl(4)#4	0.95	2.9	3.639(6)	135.5
C(11)-H(11A)···Cl(2)#5	0.95	2.65	3.556(5)	159.2
C(12)-H(12A)···Cl(2)	0.95	2.91	3.682(5)	139
C(12)-H(12A)···Cl(3)#3	0.95	2.98	3.746(5)	139
C(13)-H(13A)···Cl(4)#4	0.98	2.78	3.701(5)	157.3
C(13)-H(13C)···Cl(2)	0.98	2.86	3.542(5)	127.7
C(14)-H(14B)···Cl(3)#3	0.99	2.76	3.673(5)	153.3

Symmetry codes: #1 -1/2+*x*,1/2-*y*,1-*z*; #2 1 -*x*,-1/2+*y*,1/2-*z*; #3 3/2-x,1-*y*,1/2+*z*; #4 1 -*x*,1/2+*y*,1/2-*z*; #5 -1/2+*x*,3/2-*y*,1

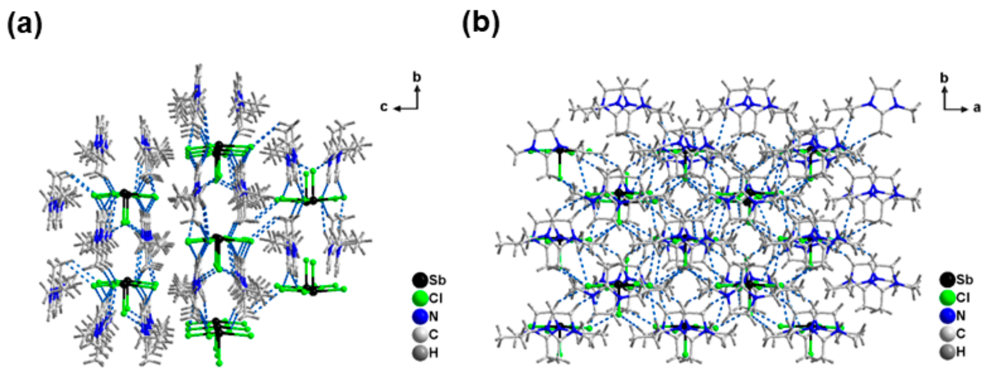

Figure S1. Supramolecular structure of compound **1** observed from *a* (a) and *b* (b) axes, respectively. Blue dotted lines represent hydrogen bond interactions.

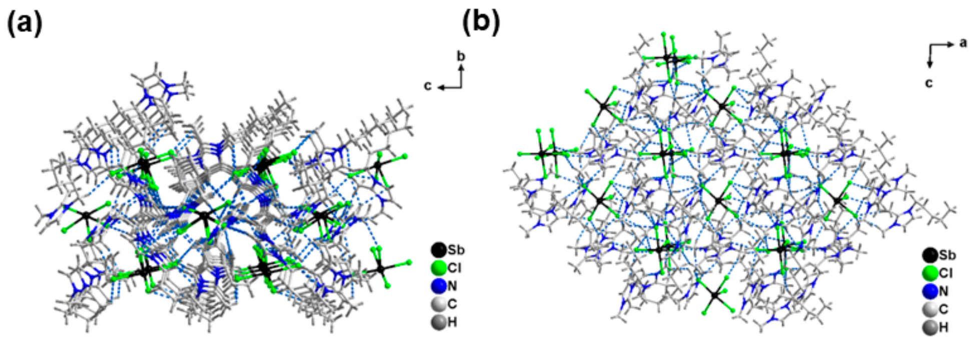

Figure S2. Supramolecular structure of compound **2** observed from *a* (a) and *b* (b) axes, respectively. Blue dotted lines represent hydrogen bond interactions.

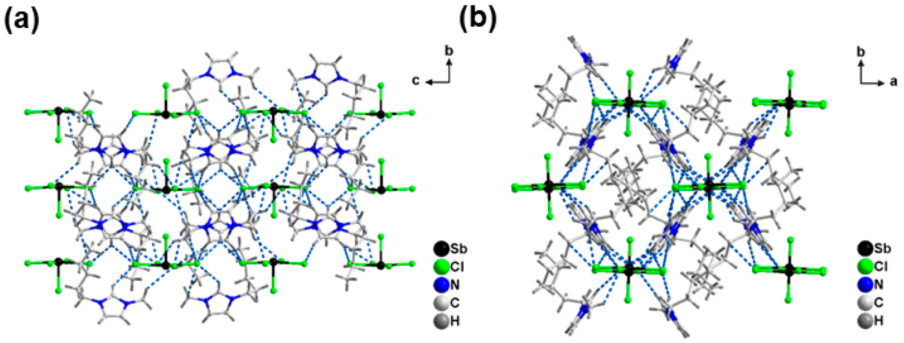

Figure S3. Supramolecular structure of compound **3** observed from *a* (a) and *b* (b) axes, respectively. Blue dotted lines represent hydrogen bond interactions.

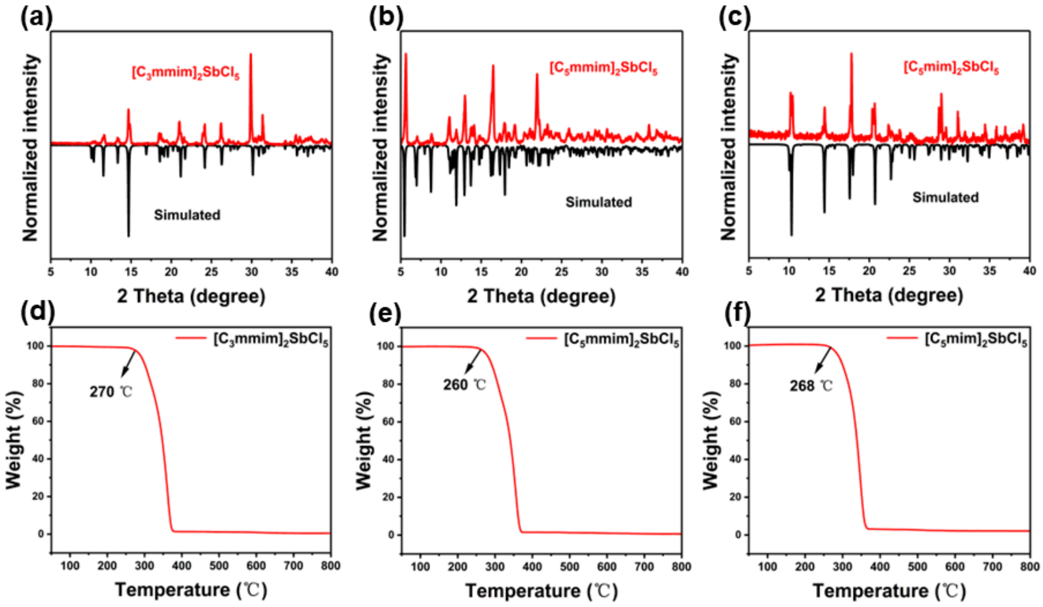

Figure S4. Experimental and simulated PXRD patterns of compounds **1** (a), **2** (b), and **3** (c). TG curves of compounds **1** (d), **2** (e), and **3** (f).

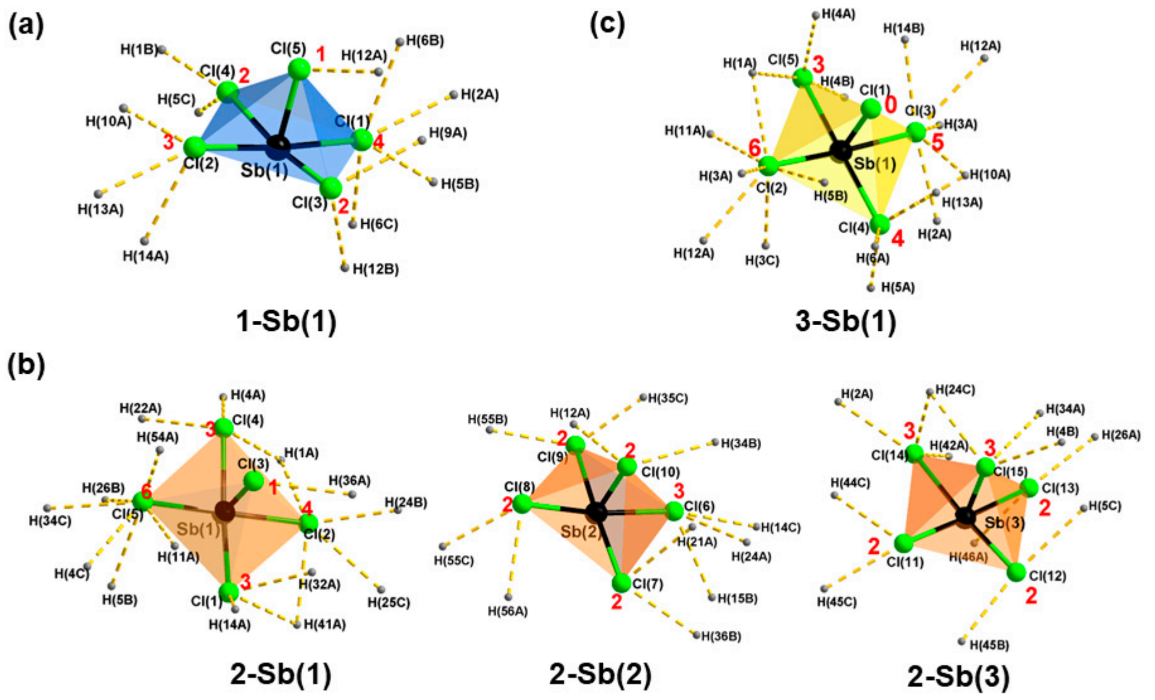

Figure S5. The hydrogen bonds between inorganic $[SbCl_5]^{2-}$ units and organic cations of compounds **1**-**3** (a-c). The red numbers present the number of hydrogen bonds around the Cl ions.

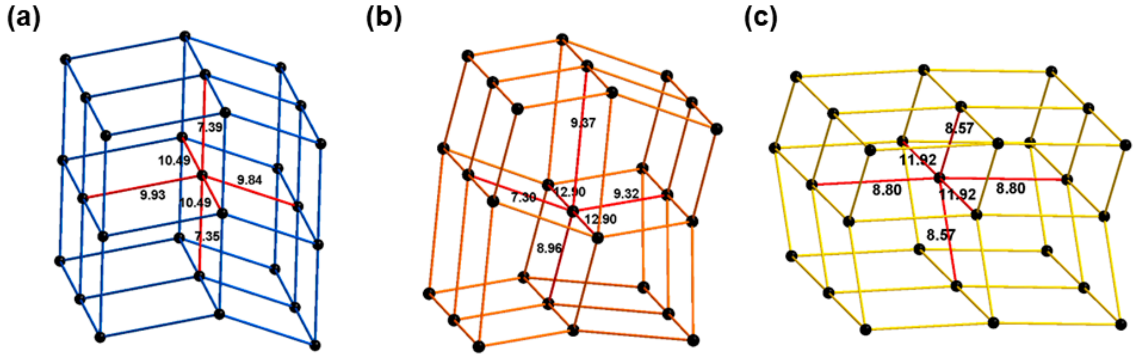

Figure S6. Diagrams showing the Sb-Sb distances (Å) between $[SbCl_5]^{2-}$ units in compounds **1** (a), **2** (b), and **3** (c). For clarity, only the Sb atoms are shown.

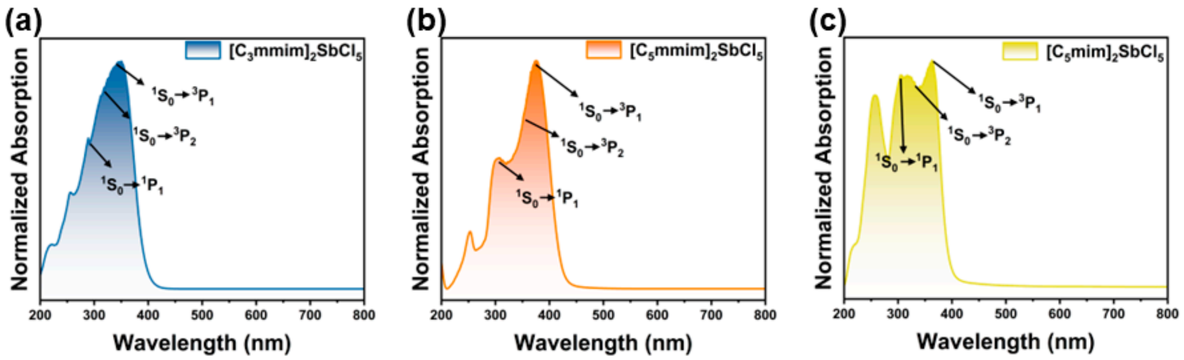

Figure S7. The UV-vis optical absorption spectra of $[C_3mmim]_2SbCl_5$ (a), $[C_5mmim]_2SbCl_5$ (b), and $[C_5mim]_2SbCl_5$ (c).

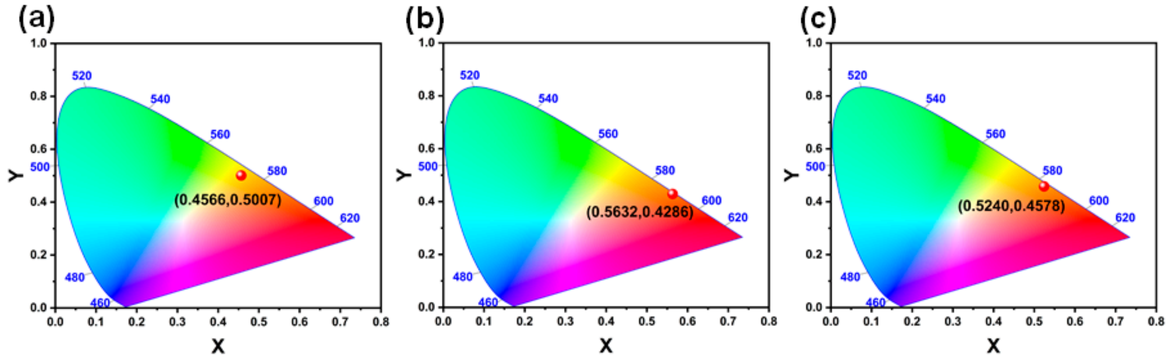

Figure S8. CIE coordinate diagrams for compounds **1** (a), **2** (b), and **3** (c).

Table S5. Summary of relevant parameters in the fluorescence lifetime fitting process

$I = I_0 + A_1 exp(-t/\tau_1) + A_2 exp(-t/\tau_2)$			
Compound	$[C_3mmim]_2SbCl_5$	$[C_5mmim]_2SbCl_5$	$[C_5mim]_2SbCl_5$
I_0	0.00166	0.00091	0.00116
A_1	0.12073	0.49423	0.05199
τ_1 (μs)	0.542	1.296	2.122
A_2	0.66384	0.3357	0.81351
τ_2 (μs)	2.545	4.035	4.111
R^2	0.9821	0.9884	0.9898
$\tau_{ave} = (A_1 {\tau_1}^2 + A_1 {\tau_1}^2)/(A_1\tau_1 + A_1\tau_2)$			
τ_{ave} (μs)	2.47	3.15	4.04

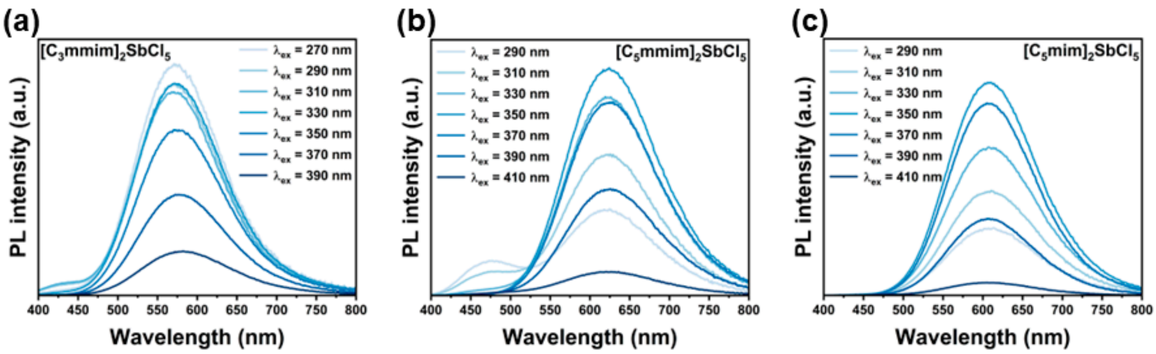

Figure S9. Emission spectra of compounds **1** (a), **2** (b), and **3** (c) under different excitations.

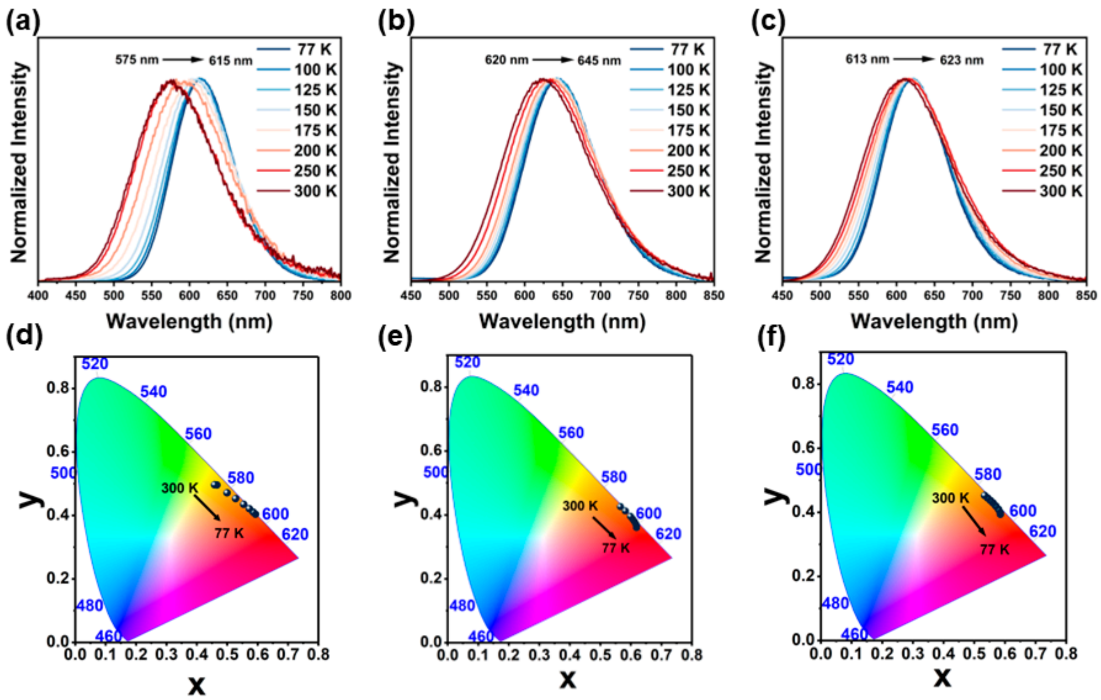

Figure S10. Normalized variable temperature spectra of compounds **1** (a), **2** (b), and **3** (c). Variable temperature CIE coordinate diagrams of compounds **1** (d), **2** (e), and **3** (f).
